# Uncovering Special Nuclear Materials by Low-energy Nuclear Reaction Imaging

**DOI:** 10.1038/srep24388

**Published:** 2016-04-18

**Authors:** P. B. Rose, A. S. Erickson, M. Mayer, J. Nattress, I. Jovanovic

**Affiliations:** 1 G.W. Woodruff School of Mechanical Engineering, Nuclear and Radiological Engineering Program, Georgia Institute of Technology, Atlanta GA 30332, USA; 2 Department of Mechanical and Nuclear Engineering, The Pennsylvania State University, University Park PA 16802, USA

## Abstract

Weapons-grade uranium and plutonium could be used as nuclear explosives with extreme destructive potential. The problem of their detection, especially in standard cargo containers during transit, has been described as “searching for a needle in a haystack” because of the inherently low rate of spontaneous emission of characteristic penetrating radiation and the ease of its shielding. Currently, the only practical approach for uncovering well-shielded special nuclear materials is by use of active interrogation using an external radiation source. However, the similarity of these materials to shielding and the required radiation doses that may exceed regulatory limits prevent this method from being widely used in practice. We introduce a low-dose active detection technique, referred to as low-energy nuclear reaction imaging, which exploits the physics of interactions of multi-MeV monoenergetic photons and neutrons to simultaneously measure the material’s areal density and effective atomic number, while confirming the presence of fissionable materials by observing the beta-delayed neutron emission. For the first time, we demonstrate identification and imaging of uranium with this novel technique using a simple yet robust source, setting the stage for its wide adoption in security applications.

The ability to effectively detect special nuclear material is one of the grand technical challenges of nuclear security that generally involves measurement of radiation with low signal to noise ratios in heavily shielded, challenging environments, for example entry ports with a requirement for two-minute scan per 40-foot container^1^,^2^,^3^. Passive detection falls short when materials of interest are shielded, requiring an external source of radiation in order to create or amplify the characteristic signatures from the material, a method referred to as active interrogation^4^. Two well-known challenges are associated with traditional bremsstrahlung-based active interrogation of shielded nuclear material: high radiation doses to cargo^5^ and vulnerability of radiation detectors to harsh radiation environments caused by the external beam^6^. The physics of bremsstrahlung-based imaging is well understood and has been demonstrated in practice^7^, including detection methods based on nuclear resonance fluorescence^8^,^9^. However, the dose associated with broad energy spectrum of bremsstrahlung and detector cost, radiation vulnerability, and particle identification issues remain to be addressed.

Two other sources of interrogating radiation have been receiving significant attention recently: the low-energy nuclear reactions^10^,^11^, in which collisions of accelerated light ions with target nuclei are used to produce gamma rays and may also produce neutrons, and the inverse Compton scattering^12^,^13^, in which laser photons are Doppler upshifted to MeV-energies in collisions with relativistic electrons. Both of those methods can produce monoenergetic or quasi-monoenergetic highly penetrating photons, opening opportunities for transmission imaging of objects with high areal density, elemental discrimination by differential attenuation, and production of unique signatures from materials that can undergo nuclear fission. Imaging potential of those two classes of sources is largely unexplored, but is of wide scientific importance beyond nuclear security, including materials science, medical imaging, low-energy nuclear physics, and industrial imaging.

Here, we present a new approach to address the challenge of detection and imaging of shielded special nuclear materials. We combine a multi-particle (high-energy monoenergetic photon and neutron) interrogation source based on a low-energy nuclear reaction – specifically, the ^11^B(d,nγ)^12^C reaction^14^–with new classes of radiation detectors designed for optimal operation in conjunction with this source. The detectors address several limitations encountered to date, including the cost of coverage for imaging of large objects, robustness while operating in high-radiation-field environments, and particle identification in mixed radiation fields. We demonstrate three favorable characteristics of this imaging method: high penetration through high-areal-density objects, elemental discrimination based on Compton scattering and pair production interaction mechanisms, and detection of nuclear materials by inducing a unique nuclear fission signature. While this work is not intended to demonstrate actual cargo scanning, we show a proof of concept of imaging and elemental characterization using a novel source based on dual-particle low-energy nuclear reaction, which is promising for development into a fully engineered scanning system. In this dual-particle approach, photons are used for imaging of the location of high-atomic number materials including SNM and shielding, while both high-energy photons and neutrons can induce nuclear fission, thereby confirming the presence of fissile or fissionable material.

## Results

### The high-energy multimodal radiation source

**Figure 1:**
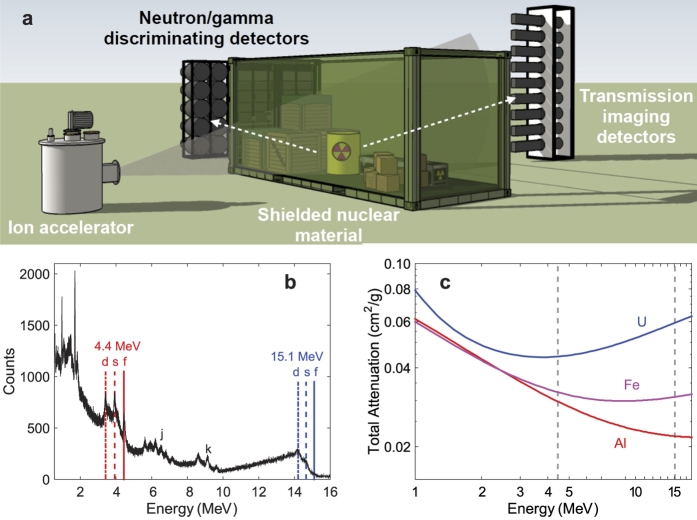
Illustration of the imaging method using a low-energy nuclear reaction radiation source. (**a**) Low-energy nuclear reaction imaging relies upon the source of monochromatic photons via a nuclear reaction between an ion accelerated to MeV-scale energy and a target. Gamma rays at discrete energies are produced from nuclear excited states of the product nucleus, with some reactions also producing neutrons. The collimated, penetrating radiation from the nuclear reaction source is used to perform transmission radiography of a shielded object, while neutron/gamma discriminating detectors detect the signature of nuclear fission. (**b**) Photon spectrum from the ^11^B(d,nγ)^12^C reaction measured with a LaBr scintillation detector. The detector is capable of measurement of the 15.1 MeV peak despite of small crystal size. It is also able to resolve the full energy peaks (labeled as “f”) and single (“s”) and double (“d”) escape peaks. Also shown are “j” and “k” peaks corresponding to other nuclear transitions in the target. (**c**) Energy-dependent attenuation for several elements (4.438 MeV and 15.1 MeV gamma energies from the ^11^B(d,nγ)^12^C reaction are shown as dashed lines).

One of the key characteristics of the low-energy nuclear reaction source is that the energies of the emitted gamma rays are governed by the nuclear structure rather than the detailed characteristics of the interaction region and interacting particles, as is the case with bremsstrahlung and inverse Compton scattering. The concept of the detection and imaging approach employed in this work is illustrated in Fig. 1a. The ^11^B(d,nγ)^12^C reaction is driven by a radio-frequency quadrupole accelerator, which produces a 3-MeV D^+^ beam with a typical current on the order of tens of μA. The D^+^ beam is incident onto a natural boron target placed inside the vacuum system of the accelerator. In addition to emission of an energetic neutron, this exothermic reaction efficiently populates the high-energy excited states of the product ^12^C nucleus, as has been shown in prior work^15^. The ^12^C nucleus subsequently emits gamma rays, with the most prominent yields at 4.438 MeV (2^+^ → 0^+^) and 15.1 MeV (1^+^ → 0^+^). Based on experimental measurements and independently characterized intrinsic efficiency of the detectors used, the gamma ray yields in our experiments were 5.3 × 10^7^ s^−1^ μA^−1^ and 6.2 × 10^6^ s^−1^ μA^−1^ at 4.438 MeV and 15.1 MeV, respectively; the fast neutron yield was 1.3 × 10^9^ s^−1^ μA^−1^. The ^12^C nucleus can also be excited to produce gamma rays by another reaction, ^12^C(p,p’)^12^C, which does not produce neutrons, but relatively high energies (~18 MeV) of the incident protons are needed to exceed the reaction threshold. In our experiment with the ^11^B(d,nγ)^12^C target, the emitted radiation is collimated into a fan beam using concrete shielding; the produced neutrons can also be efficiently removed from the beam using borated polyethylene without significantly affecting the high-energy photon flux. The examined object can be placed a variety of distances from the source, with the closest practical distance of 100 cm in our experiment. The details of experimental setup including facilities, imaging objects, detector array positions, and operating characteristics can be found in Supplementary Materials.

High penetrability and elemental specificity can be realized by energy-dependent transmission imaging^16^. Bremsstrahlung-based sources sometimes use dual- and multi-energy radiography that takes advantage of the dependence of the photoelectric (*σ*_pe_) and Compton scattering (*σ*_C_) cross sections on the atomic number *Z*; *σ*_pe_/*σ*_C_ ~ *Z*^3–4^. The choice of photoelectric absorption mechanism is partially motivated by the abundant presence of low-energy photons in the bremsstrahlung spectrum. However, low-energy photons do not have the high penetration capability needed for many applications and significantly contribute to the radiation dose. We demonstrate a compelling alternative to elemental-specific transmission imaging enabled by abundant photon flux at high energies produced by a nuclear reaction-based source (Fig. 1b). It should be emphasized that this approach is also highly suitable for the rapidly developing quasi-monoenergetic sources based on inverse Compton scattering^17^. In our method, we use the difference in atomic-number scaling of Compton scattering (σ_C_ ∝ *Z*) and pair production (σ_pp_ ∝ *Z*^2^), noting that the 4.438 MeV photon interactions are dominated by Compton scattering, whereas the 15.1 MeV photon interactions are dominated by pair production (Fig. 1c; see Fig. S1 for a more detailed representation of the energy-dependent contributions of the three main interaction mechanisms). The multi-modality of radiation produced by a reaction such as ^11^B(d,nγ)^12^C, which also produces neutrons, enables the use of a single source for neutron transmission imaging, which is highly sensitive to low-Z materials.

While the imaging alone does not provide specificity to special nuclear materials, the unique signature of prompt or beta-delayed radiation (neutrons and gamma rays from fission products) can be observed by large-coverage neutron and photon detectors. Fission can be induced by several means: neutrons produced by the nuclear reaction-based source, photofission, and neutron-induced fission by photoneutrons, since the photoneutron production cross section for the 15.1 MeV gamma ray is significant (of order 1 barn). Beta-delayed radiation is particularly promising for material detection due to much lower background in comparison to prompt radiation, which is coincident with the radiation from the active interrogation source.

### Elemental identification

**Figure 2:**
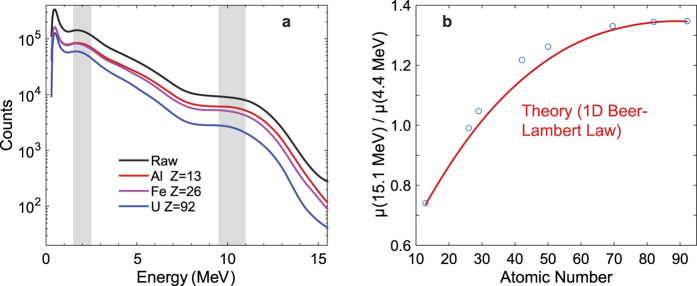
Response of Cherenkov detectors to various materials of the same aerial density. (**a**) Energy-dependent transmission measurement of several objects composed of different materials (a complete set of objects measured is provided in Supplementary Materials). The shaded regions of the spectrum are attributed to the Cherenkov detector response to 4.438 MeV and 15.1 MeV gamma rays produced in the ^11^B(d,n)^12^C reaction. (**b**) The two characteristic energy regions in (**a)** are used to reconstruct the effective atomic number of eight test objects composed of different materials. The known photon interaction cross sections for 4.438 MeV and 15.1 MeV gamma rays are used for a comparison calculation and shown as red line. The reconstruction method for the measured Cherenkov radiation spectrum and the comparison transmission calculation are described in detail in the Supplementary Materials. Error bars calculated for the measured ratios are smaller than the plot markers (circles) and are therefore not shown.

We employed a LaBr scintillation detector to measure the spectrum of photons generated by the ^11^B(d,nγ)^12^C reaction (Fig. 1b) and an array of Cherenkov detectors based on quartz^18^ to measure the energy-dependent opacity of objects of different areal density and elemental composition. Both types of detectors exhibit spectroscopic capability, and while the Cherenkov detectors have only crude energy resolution, it is sufficient for measurements when the gamma rays have large energy separation. Figure 2a demonstrates energy-dependent transmission measurement of several objects composed of different materials with the same aerial density (see Supplementary Table 1 for the details on objects measured). Since spectral analysis with Cherenkov detectors is not a common practice due to lack of resolved peaks, custom energy calibration methods using spectral shoulders and inflection points were developed^18^ specifically for this type of application. In this work the faux-peaks are integrated within bounds illustrated with shaded areas in Fig. 2a and used to determine the detected number of 15.1 MeV and 4.438 MeV photons.

Measurement of relative photon transmission at 4.438 MeV (predominantly Compton scattering) and 15.1 MeV (predominantly pair production) through materials of the same aerial density allows inferring the effective atomic number of the material. Figure 2b illustrates the decoupling of the unknown areal density *κ* from the effective atomic number *Z* by differential transmission, determined dominantly by Compton scattering or pair production. The measurement of photon transmission at two energies, *T*(*E*_1_) and *T*(*E*_2_), allows the atomic number *Z* to be extracted from the relationship between the transmission and the energy-dependent attenuation coefficients *μ*:


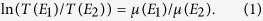


**Figure 3:**
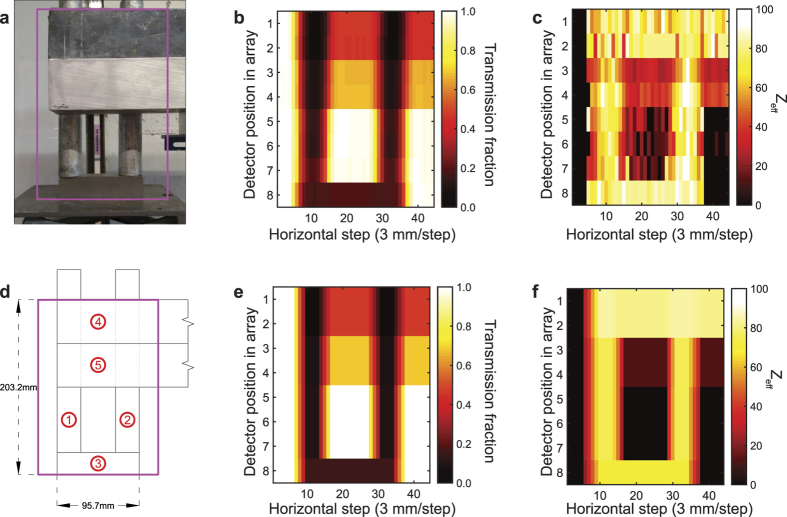
Transmission imaging of a test object placed behind 14 inch-thick borated polyethylene. (**a**) Photo of the uranium-containing object used for demonstration of transmission imaging. (**b**) Measured transmission integrated over the entire measured spectrum. (**c**) Effective atomic number, *Z*_*eff*_, reconstructed from the measured spectrum (please see Supplementary Material for details of the reconstruction). (**d**) Schematic of the object. 1 and 2–uranium rods with aluminum cladding, lead, 3 – tungsten, 4 and 5–lead and aluminum plates, respectively. (**e**) Calculated theoretical transmission of the test object for the average photon energy. (**f**) Calculated effective atomic number, *Z*_*eff*_, of the composite test object based on the known composition of the object. The indices on axes in (**b**,**c**,**e** and **f**) correspond to a step size of 25 mm in vertical direction, set by the detector pixel size (eight detectors were used in the array), and 3 mm in horizontal direction, set by the translation of the scanned object (total of 44 steps).

The experimental results are in excellent agreement with a simple one-dimensional analytical model based on the Beer-Lambert Law, showing the scaling of energy-dependent transmission *T*(*E*) = exp(−(*μ*(*E*)/ρ)*κ*), where *μ*(*E*)/*ρ* is the energy-dependent mass attenuation coefficient and *κ* is the areal density of the measured object. Determination of the atomic number then allows the areal density to be calculated from transmission at either of the photon energies. The agreement of the measurement results with the Beer-Lambert Law analytical model that uses the known mass attenuation coefficients for a range of materials studied (*Z* = 13–92) illustrates the capability to provide elemental discrimination using this method even with low-resolution detectors. A limitation of a simple two-energy approach is that it cannot readily distinguish elemental mixtures from pure elements, as it measures the “effective atomic number”, *Z_eff_*. Such ambiguities may be partially resolved by use of more than two photon energies and tomographic techniques. Monoenergetic photons at other energies can be produced from a variety of other projectiles and targets. For example, gamma rays at a range of energies up to ~18 MeV could be produced using deuteron- and proton-driven nuclear reactions such as ^27^Al(d,nγ)^28^Si, ^7^Li(p,γ)^8^Be, and ^19^F(p,α)^20^Ne. Of particular interest is the possibility of using a single projectile and interchangeable or mixed targets, which could lead to convenient practical implementations. In Fig. 3, we provide an example of imaging of a uranium-containing composite object by the energetic photons produced by the ^11^B(d,nγ)^12^C source with an array of Cherenkov detectors. We used ion chambers to measure the radiation dose due to photons in the experiment shown in Fig. 3, finding the dose to be 0.36 ± 0.016 mrem hr^−1^ μA^−1^. We provide a comparison with the American National Standards Institute (ANSI) N43.14-2011 standard^19^, which limits the dose to potential stowaways to 500 mrem (5 mSv) per scan. The low-energy nuclear reaction source delivers a dose due to photons on the order of 0.1 mrem, conservatively assuming 3-MeV D+ beam current of 1 mA and scanning time of 1 s (imaged object moving at a speed of ~3 km/h). This calculation is based on experimentally measured dose due to photons, assuming linear scaling of dose with the accelerator current. Current methods of dose assessment in conventional x-ray scanning systems involve understanding of imaging performance. Although there is no official standard for evaluating image quality of active interrogation systems, in 2008 the ANSI published the N42.46-2008 standard^20^. Four standardized tests have been described therein, including spatial resolution and penetration tests, to be used for assessing the image quality of all x-ray and gamma-ray systems used for security screening. However, these tests cannot be readily applied to systems based on monoenergetic photons: while monoenergetic systems may benefit from enhanced penetration due to larger number of high energy photons, the spatial resolution can suffer if low-dose scanning is desired. Additional dedicated studies are necessary to standardize imaging quality requirements and to evaluate the associated dose. Finally, dose due to photoneutrons may become dominant if the higher energy (10–15 MeV photons) are to be used for imaging applications.

The spatial imaging resolution in this measurement is 25 mm in vertical direction, set by the detector pixel size used, and 3 mm in horizontal direction, set by the step size in the translation of the scanned object. The spatial resolution can be readily improved by increasing the detector coverage and by using finer step size in translation of the scanned object. The image of the object shown in Fig. 3a was obtained from the total photon beam transmission through the object across the entire energy spectrum. Spatially dependent *Z*_*eff*_ of the imaged object (Fig. 3b) was reconstructed by the differential transmission method illustrated in Fig. 2. The experimentally measured energy-integrated beam transmission is in excellent agreement with the calculated transmission (see Supplementary Materials for details) based on a simple 1-D Beer-Lambert Law model (Fig. 3e). Similarly, the reconstructed *Z*_*eff*_ is in a good agreement with the effective atomic number present in the composite object (Fig. 3f), enabling spatially resolved material identification. We note that the atomic number discrimination among high-*Z* materials using transmission measured at 4.438 MeV and 15.1 MeV is challenging, as the sensitivity of the ratio of the attenuation coefficients at those two energies to atomic number *Z* is low for 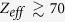 (Fig. 2b).

### Confirmation of the presence of fissile or fissionable material by delayed neutron emission

**Figure 4:**
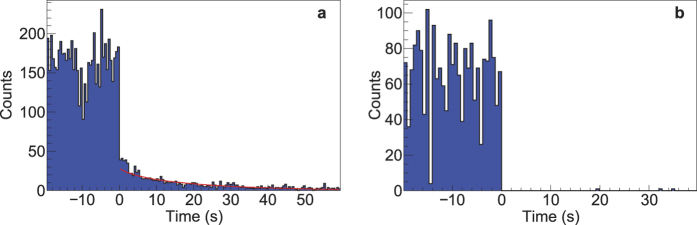
Observation of emission of beta delayed neutrons as a unique signature of material undergoing nuclear fission. The interrogating beam is turned off at time = 0 s. (**a**) Temporal profile of delayed neutrons with a natural uranium target observed using a low-threshold composite fast neutron detector is in good agreement with a common parameterization into six delayed neutron groups (red line). (**b**) Temporal profile of delayed neutrons with a tungsten target using a low-threshold composite fast neutron detector shows no emission of delayed neutrons. More information on the neutron measurements is provided in Supplementary Materials.

Independent measurement of material areal density and *Z*_eff_ may not be sufficient to detect the presence of elements and specific isotopes that could be used as nuclear explosives (^233^U, ^235^U, and ^239^Pu), as illustrated in Fig. 3c. A unique signature of fissionable materials is the emission of characteristic prompt and delayed radiation (neutrons and gamma rays). Both the neutrons and the 15.1 MeV gamma ray produced in the ^11^B(d,nγ)^12^C reaction are suitable for inducing nuclear fission. Prompt radiation produced in fission is challenging to detect due to the high intensity of background; in the case of the ^11^B(d,nγ)^12^C reaction this includes the gamma rays and neutrons emitted by the source itself and photoneutrons produced in the surrounding materials, especially higher *Z* materials. This motivates the detection of delayed neutrons and gamma rays, which also exhibit a characteristic decay profile. To augment the imaging, we performed an experiment using natural uranium (99.3% ^238^U, 0.7% ^235^U) irradiated by the nuclear reaction source, in which fission was induced predominantly by neutrons. The delayed neutron emission is coincident with the abundant emission of photons originating from activation of surrounding material and from fission products, if fission is induced. Separation of beta-delayed neutrons, which have relatively low energy (mean energy of ~0.5 MeV prior to any thermalization in the surrounding medium), from the photon background can be challenging. We realized this separation at a low energy threshold by a novel class of neutron detector based on a composite of Li-doped glass and scintillating plastic, which relies on capture gating together with pulse height and pulse shape discrimination^21^. In Fig. 4a,b, we present the detected delayed neutron signature from natural uranium and tungsten, respectively, demonstrating the capability of the ^11^B(d,nγ)^12^C reaction to simultaneously provide a suitable probe for discovery of special nuclear material, if the beta-delayed radiation can escape the material shielding. We note that the emitted neutrons and 15.1 MeV photons, along with the photoneutrons produced in the object and the object’s surroundings, can temporarily activate the materials, enabling another mode for achieving elemental and isotopic specificity via the well-developed techniques of neutron activation analysis^22^ and photon activation analysis^23^.

## Discussion

We demonstrated a novel modality of imaging that employs a source of photons and neutrons based on low-energy nuclear reactions. The high energy of the produced photons and the multi-modality of the source enables dual and multi-energy transmission radiography as a method to achieve contrast among different elements, while simultaneously inducing fission signatures needed to confirm the presence of materials that could be used in construction of a nuclear explosive. It has been shown that the high-rate Cherenkov detectors are suitable for transmission measurements and that their limited resolution does not present an obstacle to material identification by differential transmission. Low-threshold composite neutron detectors have been shown to produce strong rejection of the abundant photon background, thereby permitting detection of material undergoing nuclear fission. Both Cherenkov and composite neutron detectors are low-cost, offering opportunities for practical wide-coverage imaging. This approach to active interrogation is highly flexible, since a variety of nuclear reactions can be chosen to generate monoenergetic photons with optimal energies for a range of imaging problems. In addition, the source is robust; while the photon and neutron yields can be greatly dependent on the reaction target purity and design, as well as the incident ion beam energy, the resulting gamma ray energies are set by the excited state of the product nucleus. These characteristics of the nuclear reaction source, combined with optimized radiation detectors and reconstruction techniques, could offer significant advances in detection of shielded special nuclear materials.

## Methods

### The ^11^B(d,nγ)^12^C source

The ^11^B(d,nγ)^12^C source is driven by a modified LANSAR Model DL-3 radiofrequency quadrupole accelerator manufactured by Accsys Technology Inc. and located at the Massachusetts Institute of Technology Bates Linear Accelerator Center (Middleton, Massachusetts). The accelerator produces a 3-MeV D^+^ beam with varying pulse rates and widths, delivering an average current of up to 90 μA at up to 800 Hz repetition rate with a duty cycle of up to 1.6%. Natural boron was used as a target, with an approximate ^10^B isotopic abundance of 19.9% and ^11^B isotopic abundance of 80.1%. The thickness of the natural boron target was 2.0 mm. The target was surrounded by borated polyethylene, concrete, and lead shielding to produce a fan beam with a divergence angle of 8.62 mrad (horizontal) and 276 mrad (vertical). Two classes of probes were used: (1) photon probe, when the neutrons produced by the target were shielded, and (2) multi-particle (photon and neutron) probe, when the target neutron shielding in the beam direction was removed. The target shielding and collimation setup for those two types of probes is shown in Supplementary Fig. S2.

### Measured objects

The transmitted spectrum of eight objects was measured. The objects, located at position (1) in Supplementary Fig. S2, were composed of different pure elements with a range of atomic numbers (*Z* = 13–92) and thicknesses (Supplementary Table S1). After the beam was transmitted through an object, it was collimated by layers of concrete blocks, as well as iron and lead bricks. All objects chosen for the experiment had an approximate areal density of 19.5 g/cm^2^, except for the tungsten object, which had an areal density of 14.5 g/cm^2^. The densities were measured for all materials by measuring their dimensions and masses. From these measurements it was determined that the tungsten sample was not pure, but rather that it was a common copper tungstate alloy of 90% tungsten and 10% copper, also known as CuW90, which yields *Z*_*eff*_ = 69.5.

Depleted uranium plates with two standard sizes (200 × 200 × 0.79 mm^3^ and 105 × 105 × 3.05 mm^3^) were used for the uranium transmission measurement. Ten natural cylindrical uranium rods encapsulated in aluminum cladding (diameter = 27.52 mm, length = 238.12 mm, *m* ~ 2 kg) were used to study the emission of beta-delayed radiation. The design of the object used to demonstrate transmission imaging of shielded uranium is shown in Fig. 2(a,d) and included a 15.95 mm-thick lead region, a 52.22 mm-thick aluminum region, and a bare protrusion. Two natural uranium rods were placed behind these materials and on top of a 25.60 mm-thick tungsten block, forming a shape resembling the letter “U”.

### Gamma ray detectors

Gamma energy spectrum was acquired with a 1.5-inch LaBr scintillation detector, model LABR-1.5 × 1.5, manufactured by Canberra Industries. Energy calibration was performed using the known photopeaks at 3.4, 3.9, 4.4, 14.1, 14.6, and 15.1 MeV. Three Cherenkov detectors were constructed and used in this study: (1) 2-inch long quartz crystal coupled to a Hamamatsu R292 photomultiplier tube (PMT) with quartz window and sensitivity limit of 190 nm; (2) 4-inch long cylindrical quartz crystal coupled to a Hamamatsu R6095-03 PMT with a sensitivity limit of 300 nm; and (3) an array of six 6-inch long quartz crystals coupled to Hamamatsu R374 PMTs with UV transmitting glass windows and a sensitivity limit of 185 nm. The windows of the R292 and R374 PMTs have a refractive index very similar to that of quartz, reducing the light loss on the boundary. Since the Cherenkov radiation is more intense in the blue/UV region, the R6095-03 PMT cuts off the signal below approximately 300 nm, reducing the detector light output compared to other designs. All Cherenkov detector crystals were cylindrical in shape, with a 25 mm diameter. During the analysis, the detector configuration was taken into account in order to compare the signal among different designs. Energy calibration methodology was developed specifically for Cherenkov detectors^18^.

### Neutron detectors

Two types of neutron detectors were used to measure the emission of beta-delayed neutrons from uranium: the liquid scintillation detector EJ-309 (Eljen Technologies) and a specially designed composite scintillation detector based on Li-doped scintillating glass rods embedded in a scintillating polyvinyltoluene matrix^21^.

The EJ-309 detectors are based on nuclear (primarily hydrogen) recoil, and exhibit pulse shape discrimination that can be used to separate the scintillation pulses produced by photons from those produced by neutrons. This discrimination is effective only at higher proton recoil energies. The EJ-309 detector active volume had the dimensions of 50.8 mm diameter x 50.8 mm height and was interfaced to a photomultiplier tube with a 52-mm diameter cathode (Hamamatsu R7724). It has been found that the low energy of delayed neutrons prevented their effective separation from the abundant gamma background after the accelerator beam was turned off. Therefore, the remaining measurements were made with specialized composite neutron detectors.

The composite neutron detector relies on neutron thermalization and capture to discriminate neutrons from photons, with the misclassification fraction <10^−8^ demonstrated with a radioisotope neutron source. The active volume was cylindrical, with a diameter of 50 mm and height of 50 mm; detailed detector design and performance has been described in prior work^21^. The scintillation active volume was coupled to a fast photomultiplier tube (Hamamatsu R6231-100, 46-mm diameter). Due to its relatively small size and detection mechanism, this detector is more effective in detecting lower energy neutrons, making it well suited for beta-delayed neutron measurements. The typical energy-pulse shape parameter performance in the experimental area used is shown in Supplementary Fig. S3.

### Data acquisition, analysis, and simulation

The signals produced by photon and neutron detectors were digitized using 14-bit, 500-MHz, 8-channel desktop digitizers (CAEN DT5730). In delayed neutron measurements, a custom data acquisition suite was used, capable of digitizing 600-ns long waveforms at rates of up to 14 kHz. Full digital forms were stored and later used in the analysis. The waveforms were time stamped to allow reconstruction of the beta-delayed neutron emission time profile.

In gamma ray transmission and imaging studies, the digitizer on-board field-programmable gate array (FPGA) was used to process and analyze the digitized waveforms in real time. The built-in firmware for pulse shape discrimination (PSD) and FPGAs were used to read out processed data in the form of energy per pulse. This method minimizes the data transfer rate between the digitizer and control software, which allows for the use of many detectors in parallel with few computational resources. In Cherenkov detectors the speed of the PMT determines the speed of the entire detector. With the FPGAs, we able to acquire and process a complete pulse every 20 ns per detector.

The data analysis was carried out using the open-source data analysis suite ROOT^24^. Digital data processing is based on standard methods of waveform baseline subtraction, and pulse area integration, which were previously described^18^,^21^ for photon and neutron detectors, respectively.

Monte Carlo simulations of target yields and the interaction of photons and neutrons with the test objects, detectors, and shielding were carried out using the Geant4.10 simulation framework^25^. The dose calculation was performed using the Monte Carlo framework MCNP6^26^. Nuclear reaction data was retrieved from the Evaluated Nuclear Data File ENDF/B-VII.1^27^, and the photon cross section from the NIST XCOM database^28^.

### Measurement protocol

Transmission measurements were performed with the accelerator current of 21.0 μA. The integration time for each object was 2700 s and the measurements were performed with borated polyethylene shield in place. Test objects were placed at a distance of 140 cm from the source (boron target) to the centerline of the object, along the axis defined by the accelerator D^+^ beam. The horizontal beam size at the location of a test object was much wider than the test object, but all test objects were much wider than the collimated beam width between the test object and the detectors. The Cherenkov detectors were located approximately 770 cm from the test objects and separated by multiple layers of collimation, so that the build-up effects arising from interaction with the test objects were negligible. The total distance from the boron target to the detectors was 909 cm. The projected width of the beam on the detectors was 8.0 cm, which exceeds the width of the detectors (2.5 cm).

Measurements of beta-delayed neutrons were conducted by placing the two types of neutron detectors (EJ-309 and composite detector) in the immediate vicinity of the U target (at an average distance of ~10 cm). For neutron measurements, the borated poly shield was removed to permit the neutrons produced in the ^11^B(d,nγ)^12^C reaction to reach the test object. The accelerator current was set to 23 μA. Irradiation was performed for 300 s, at which point the accelerator beam was shut off and neutron measurements were conducted, with an integration time of 900 s. Three identical measurements were performed in this manner, and the data were summed to improve the statistics of the measurement.

The dose rate delivered to the imaged material was measured in the beam line. These measurements were conducted under the same shielding conditions as the imaging experiments, *i.e.* with 14 inches of borated polyethylene in place. The gamma dose was measured by an ion chamber manufactured by Fluke Biomedical, Inovision model 451P. The neutron dose measurement was conducted with a NRD 9 inch neutron ball containing a ^10^B proportional counter and manufactured by Thermo Scientific Corporation. Both dose meters were operated in integration mode and run separately for 5 minutes each in the beam line. Background measurements were taken immediately after accelerator operation to account for any potential activation products in the experimental set up.

## Additional Information

**How to cite this article**: Rose, P. B. *et al.* Uncovering Special Nuclear Materials by Low-energy Nuclear Reaction Imaging. *Sci. Rep.* **6**, 24388; doi: 10.1038/srep24388 (2016).

## Supplementary Material

Supplementary Information
