# Supplementary material for: Uncovering Special Nuclear Materials by Low-energy Nuclear Reaction Imaging
Source: Sci Rep. 2016 Apr 18;6:24388. doi: 10.1038/srep24388 (PMC4834544; doi:10.1038/srep24388)
Supplement: Supplementary Information [file srep24388-s1.pdf]

# Uncovering Special Nuclear Materials by Low-energy Nuclear Reaction Imaging

## Supplementary Materials

P. B. Rose Jr.[1], A. S. Erickson[1,*], M. Mayer[2], J. Nattress[2,†], I. Jovanovic[2,*,†]
[1]G.W. Woodruff School of Mechanical Engineering, Nuclear and Radiological Engineering Program, Georgia Institute of Technology, Atlanta GA 30332, USA
[2]Department of Mechanical and Nuclear Engineering, The Pennsylvania State University, University Park PA 16802, USA

*Correspondence to: Anna Erickson (erickson@gatech.edu) and Igor Jovanovic (ijov@umich.edu)

†Current address: Department of Nuclear Engineering and Radiological Sciences, University of Michigan, Ann Arbor MI 48109, USA

### Extended data figures and tables

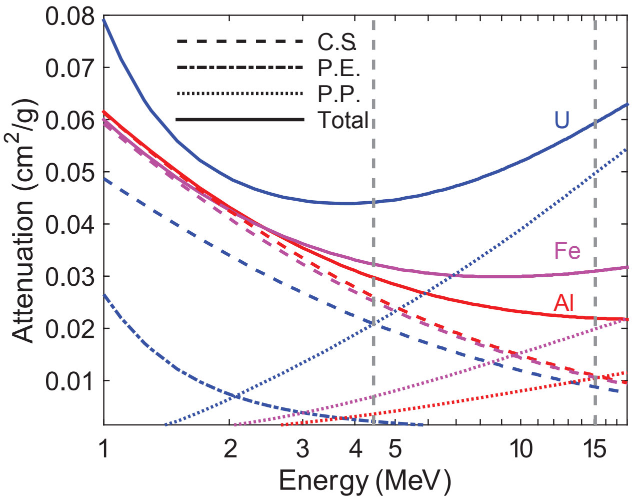


**Figure S1 |** Individual contributions from photoelectric (P.E.), Compton scattering (C.S.), and pair production (P.P.) to the total photon attenuation cross section (Total).

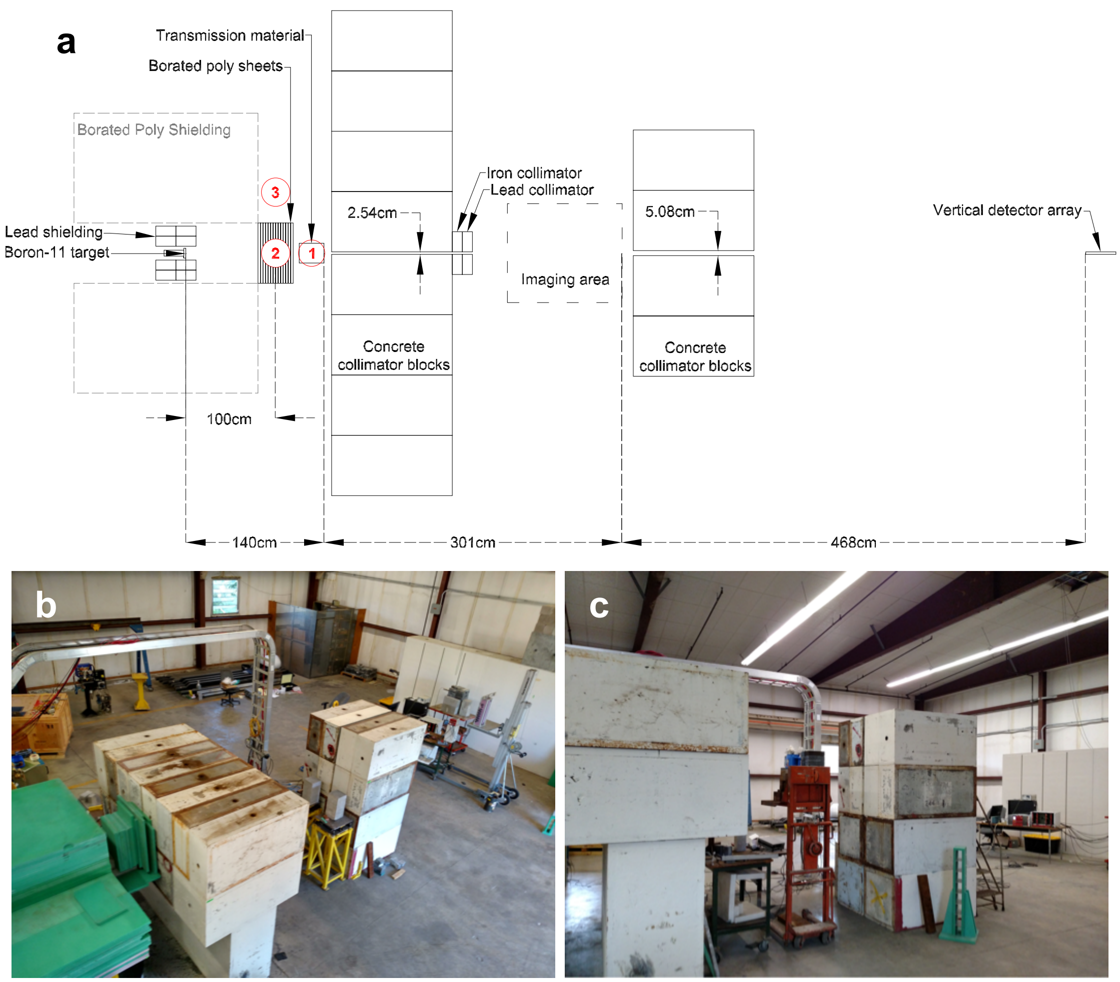


**Figure S2 | (a)** Schematic of the shielding and collimation setup for the experiment. The position (1) is the location at which test objects are inserted for gamma transmission measurements. The position (2) represents the location of the borated polyethylene shield, which is replaced with natural uranium test objects for measurements of beta-delayed neutrons. The position (3) is the location at which the neutron detector is placed for beta-delayed neutron measurements. (**b**) Top view of the concrete collimator blocks (122 cm thick in the direction of the beam) and borated polyethylene sheets used to shield the $^{11}B(d,n\gamma)^{12}C$ source. (**c**) Side view of the concrete collimator blocks.

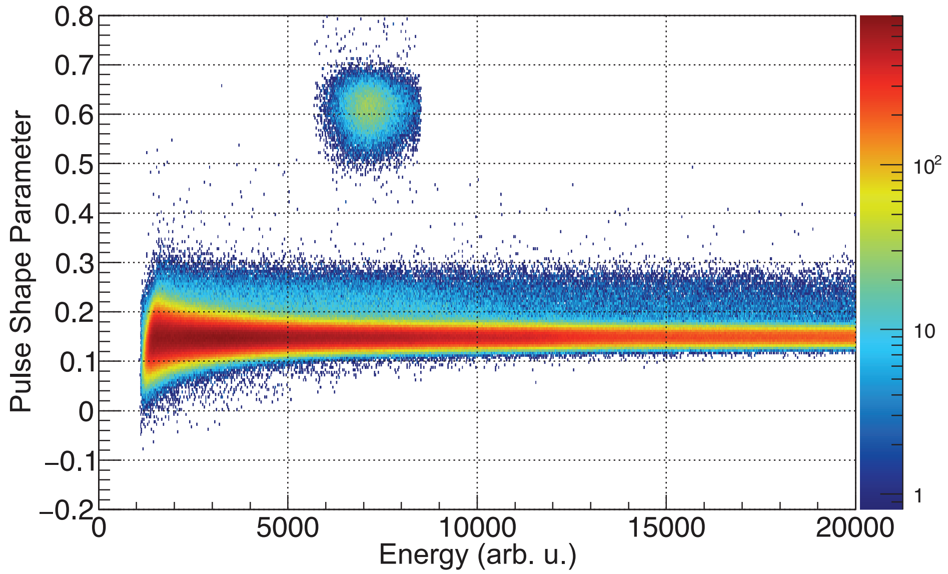


**Figure S3 |** Composite detector performance in the experimental area while the $^{11}B(d,n\gamma)^{12}C$ source is in operation. Neutrons are effectively separated from photons in the region of Pulse Shape Parameter of approximately 0.5–0.7 and energy of approximately 6000–9000 arb. u.

Table S1. Objects tested for transmission. The atomic number of W corresponds to the $Z_{eff}$ of the copper tungstate alloy.

| Material | *Z* | Thickness (mm) | Calculated Transmission | |
|---|---|---|---|---|
| | | | 4.438 MeV | 15.1 MeV |
| Al | 13 | 71.05 | 59.7% | 66.9% |
| Fe | 26 | 24.62 | 57.2% | 56.3% |
| Cu | 29 | 21.40 | 54.5% | 52.0% |
| Mo | 42 | 19.54 | 52.8% | 45.0% |
| Sn | 50 | 26.56 | 53.3% | 44.2% |
| W | 69.5 | 8.81 | 58.7% | 48.2% |
| Pb | 82 | 19.29 | 41.5% | 30.1% |
| U | 92 | 10.16 | 41.3% | 29.9% |

## Supplementary Note 1: Calculation of the effective atomic number, $Z_{eff}$

The effective atomic number of an object composed of multiple pure elements or a mixture of elements can be defined by considering a hypothetical pure element object with an areal density equal to that of the composite object, such that the pure element object produces the same photon attenuation. For an object containing $i$ components, each with mass attenuation coefficient $\mu_i/\rho_i$ and areal density $\kappa_i$, we can write

$$T = \prod_i \exp(-\mu_i/\rho_i\kappa_i) = \exp(-\sum_i \mu_i/\rho_i\kappa_i) = \exp\left(-\left(\frac{\mu}{\rho}\right)_{eff}\kappa\right), \quad (1)$$

where $T$ is the photon transmission and $\kappa = \sum_i \kappa_i$ is the total areal density. $Z_{eff}$ can then be defined as the atomic number of the element most consistent with the mass attenuation coefficient $\left(\frac{\mu}{\rho}\right)_{eff}$.

## Supplementary Note 2: Determination of the photon and neutron flux

There is a considerable disagreement in the reported yields from the $^{11}B(d,n\gamma)^{12}C$ reaction in the literature[11,15,29], which has been attributed both to the composition of the target and the method of data acquisition (limits set on time gating). In our experiment we used a natural boron target significantly thicker than the range of 3-MeV $D^+$ particles in boron (48 μm, calculated using the SRIM code[30]).

We calculated the photon flux from the detected rates in a LaBr detector, the distance from the source, and known intrinsic efficiency extrapolated to the higher energy. Those results are also summarized in Table S2. The fast neutron production rate was calculated from the detected delayed neutron rate, known fast neutron-induced fission cross section, independently measured and calculated neutron detector efficiency[21], and the known test object material (natural uranium), mass, and geometry. The result is in good agreement with the work of Taddeucci *et al.*[11].

Table S2. Photon and neutron production rate from the $^{11}B(d,n\gamma)^{12}C$ reaction, normalized to 1 μA current

| Method | 4.438 MeV gamma ($s^{-1}$) | 15.1 MeV gamma ($s^{-1}$) | Fast neutrons ($s^{-1}$) |
|---|---|---|---|
| LaBr detector | $5.3 \times 10^7$ | $6.2 \times 10^6$ | – |
| Neutron detector | – | – | $1.3 \times 10^9$ |

## Supplementary Note 3: Parametrization of beta-delayed neutron emission

The neutron flux detected by the composite neutron detector was compared to the beta-delayed neutron emission. The neutron flux was plotted as a function of time and regions where the interrogating beam was on and off was identified. The detected rate $R(t)$ of beta-delayed neutrons was fitted to the six delayed neutron groups, corresponding to six characteristic decay constants. The fitting function used was

$$R(t) = B + C\sum_{i=0}^{6}\frac{\varepsilon_i Y_i}{\tau_i}e^{(-t/\tau_i)}, \quad (2)$$

where $B$ is the background, $C$ is a scaling constant, index $i$ is the group number, $\varepsilon_i$ is the detector efficiency for group $i$, and $Y_i$ is the beta-delayed neutron yield per fission for group $i$. The parameters used correspond to fast fission of $^{238}U$ and are listed in Table S3. For a fit of the detected neutron rate over the sixty seconds immediately after the accelerator beam was turned off, the values of $B$=0.1051 and $C$=0.5145 gave a $\chi^2/dof$=1.028.

Table S3. 6-group beta delayed neutron parameters used in fitting the detected delayed neutron rate[31]

| Group | Y | $\varepsilon$ | $\tau$ (s) |
|---|---|---|---|
| 1 | 0.00054 | 0.015894 | 52.38 |
| 2 | 0.00564 | 0.010782 | 21.58 |
| 3 | 0.00667 | 0.012398 | 5.00 |
| 4 | 0.001599 | 0.01231 | 1.93 |
| 5 | 0.00927 | 0.013507 | 0.490 |
| 6 | 0.00309 | 0.011723 | 0.172 |

## Supplementary Note 4: Magnitude of delayed neutron signature and relationship to dose

Inducing the delayed neutron signature requires irradiation, that results in a radiation dose delivered to a possible stowaway. Here we estimate the required dose that induces a delayed neutron signature of sufficient magnitude to be detectable. In our experiment fission was induced primarily by fast neutrons. We calculate the magnitude of the delayed neutron signature based on the neutron production rate of $3.1\text{x}10^{10}$ $s^{-1}$ in the $^{11}B(d,n)^{12}C$ reaction for the $D^+$ current of 24 μA, the mass of the natural uranium object of ~20 kg, the object geometric configuration in the beam, distance of the uranium object from the neutron source of 100 cm, and the approximate time to achieve the buildup of delayed neutron emission of 20 s. The incident neutron rate on the uranium test object is $5.9\text{x}10^7$ n $s^{-1}$, and the incident neutron flux at the object is $2.5\text{x}10^5$ n $cm^{-2}$ $s^{-1}$. The measured delayed neutron rate at the detector at the time the accelerator beam is turned off is 27 n $s^{-1}$, and the total number of neutrons measured over 50 s from the time the accelerator beam is turned off is 327. We use an intrinsic capture efficiency of 1% for delayed neutrons in the detector and average distance of the neutron detector from the uranium test object of 10 cm. We calculate the delayed neutron rate in natural uranium to be initially $1.5\text{x}10^3$ n $s^{-1}$ $kg^{-1}$ and a total of $1.8\text{x}10^5$ n $kg^{-1}$ produced over 50 s from the time the accelerator beam is turned off. These rates correspond to $1.2\text{x}10^{-5}$ n $s^{-1}$ and $1.6\text{x}10^{-4}$ n per source neutron delivered over the 20 s period immediately preceding the delayed neutron measurement.

We next estimate the dose rate to a stowaway. The entire fast neutron spectrum produced in the experiment was not measured and is dependent on the incident deuteron energy. For this calculation we assume an average neutron energy from the $^{11}B(d,n)^{12}C$ source to be 5 MeV, which produces a dose to soft tissue[32] of $4.1\text{x}10^{-9}$ rad $cm^2$.

Let us assume that the total number of neutrons needed to detect 25 kg of uranium in a practical, efficient system is $10^3$. On the basis of the above analysis we estimate the dose of 50 mrad to stowaway over 20 s of neutron irradiation to produce this signature.

We note that significant reductions in dose may be achieved by measurement of the neutron buildup if an interrogating source exhibits a time structure that allows delayed

neutron rate to be measured between the source pulses. This reduction would result from measuring both the buildup and the decay of the delayed neutron rate.

One challenge that must be addressed in these measurements is the natural fast neutron background. This background can be rejected by two methods: (1) subtraction, due to its constant rate, and (2) energy discrimination in a composite capture-gated neutron detector that enables the measurement of neutron thermalization yield, since the mean energy delayed neutrons is significantly lower than that of fast neutron background.

## Supplementary Note 5: Reconstruction of image and $Z_{eff}$ from measured transmission

The image in Fig. 4b represents the fraction of number of the number of photons transmitted through the object, integrated over the entire measured energy spectrum. The index of the Cherenkov detector is shown on the vertical axis, while the index of the scanning step is shown on the horizontal axis. The test object was translated in 3 mm steps, yielding a matrix of 8x44 pixels. Each row of the matrix represents measurements taken by the same detector. The measurement time for each pixel was 5 minutes. The energy spectrum measured at each pixel was integrated to obtain the total number of photons detected at that pixel by the Cherenkov detectors. The relative transmission at each pixel was obtained by normalizing the measured spectrum with the object in the beam to the spectrum measured with the object removed from the beam (average of the first four pixels shown in Fig. 4b). This approach allows for elimination of effects of any differences between the eight Cherenkov detectors from the image.
Dual-energy differential transmission measurement at 4.438 MeV and 15.1 MeV has been used to reconstruct the effective atomic number, $Z_{eff}$, shown in Fig. 4c. A simplified assumption is made that any interaction of the photon in the object removes the photon from the beam, so that it does not reach the detector. For two energies $E_1$ and $E_2$, in the transmission measurement we have

$$T(E_{1,2}) = I(E_{1,2})/I_0(E_{1,2}) = \exp\left(-(\mu/\rho)_{1,2}\kappa\right) \qquad (3)$$

and the following ratio can be constructed:

$$\frac{\ln T(E_1)}{\ln T(E_2)} = \frac{(\mu/\rho)_1}{(\mu/\rho)_2} \qquad (4)$$

$Z_{eff}$ is subsequently chosen as the atomic number for which the ratio of mass attenuation coefficients at energies $E_1$ and $E_2$ most closely matches that obtained from equation (4).
Fig. 4e and 4f were calculated from the known material geometry and composition, discretized onto an 8x44 grid. The transmission ratio shown in Fig. 4e was calculated by first finding a yield-weighted average energy of the 4.438 and 15.1 MeV photons (reported in Table 2) of 5.52 MeV. We then use the NIST XCOM database[28] attenuation coefficients at this average energy for each material to calculate a total transmission using the Beer-Lambert law. In this calculation we use single photon energy and calculate total attenuation corresponding to all known material at a given pixel. The transmission at the

average photon energy is shown for comparison with Fig. 4b. Fig. 4f was produced by estimating the average thickness of each material in a pixel, and then calculating $Z_{eff}$ a photon encounters propagating through the pixel.
